# Efficacy of iguratimod in the treatment of patients with palindromic rheumatism ineffective to methotrexate or hydroxychloroquine

**DOI:** 10.3389/fimmu.2025.1613129

**Published:** 2025-06-16

**Authors:** Mengjie Chen, Yongjun Cheng, Guiyao Jin, Yuren Tu, Qi Zhang, Chuanfu Zhang, Wenlong Wang

**Affiliations:** ^1^ Department of Rheumatology and Immunology, The First People’s Hospital of Wenling, Wenling, China; ^2^ Department of General Surgery, Yuhuan Second People’s Hospital, Yuhuan, China; ^3^ Department of Rheumatology and Immunology, Seventh People’s Hospital of ShangHai University of Traditional Chinese Medicine, Shanghai, China

**Keywords:** arthritis, treatment, DMARD, palindromic rheumatism, iguratimod

## Abstract

**Introduction:**

Palindromic Rheumatism (PR) is a rare form of arthritis characterized by recurrent episodes of joint and periarticular inflammation. Given the paucity of established treatment guidelines due to its rarity and complex pathogenesis, we aimed to analyze the efficacy and safety of iguratimod (IGU) in the treatment of refractory PR.

**Methods:**

This retrospective study included patients with PR who attended the First People’s Hospital of Wenling between January 2019 and September 2023. 32 patients with poor response to methotrexate (MTX) and hydroxychloroquine (HCQ) were enrolled and were switched to IGU 25 mg twice daily alone or in combination with MTX 10 mg weekly. The primary outcomes measured included the frequency and duration of disease attacks over a three-month period. Complete remission was defined as no attacks within three months, partial remission as a reduction of at least 50% in attack frequency, and no remission as less than a 50% reduction.

**Results:**

The median treatment duration with IGU was 11.3 months. The results demonstrated a significant reduction in the number of attacks over a three-month period (1.3 ± 1.4 vs. 5.8 ± 2.0, P < 0.0001). Furthermore, patients experienced a decrease in attack frequency and an increase in remission duration (78.0(33.8,99.0) days vs. 15.0(13.0,22.0) days, P < 0.0001). The duration of each attack was also shortened (2.1 ± 0.7 days vs. 2.5 ± 0.8 days, P=0.0042). Only one patient discontinued IGU due to gastric upset.

**Conclusion:**

Iguratimod has demonstrated favorable efficacy and safety in the treatment of patients with PR who have not responded adequately to MTX and HCQ, which needs to be further confirmed.

## 1 Introduction

Palindromic rheumatism (PR) is a specific type of arthritis characterized by recurrent episodes of swelling and pain in the joints and periarticular structures, usually in the form of wandering monoarticular episodes that last from a few hours to a few days and resolve without residual joint damage (1). Similar to rheumatoid arthritis (RA), the most commonly affected joints are the wrist, metacarpophalangeal joints and proximal interphalangeal joints (1). In different cohort studies, the percentage of patients with PR who progressed to RA ranged from 27.5% to 66.7%, especially in patients with positive anti-cyclic citrullinated peptide (anti-CCP) antibodies (2–5). Based on the shared genetic and immunological risk factors, as well as typical joint involvement and natural disease progression, it was once supposed that PR might be an early rheumatoid arthritis (1). However, a clinical phenotype of intermittent self-limiting attacks and the distinct imaging phenotype of reversible non-synovial extracapsular inflammation often without synovitis suggest that PR may be an independently existing autoinflammatory disease (6, 7). This is further strengthened by a recent whole-exome sequencing study demonstrating that PR and RA are not genetically similar (8).

Due to the relative rarity of PR and the complex scenario of its pathogenesis, there are no accepted treatment guidelines, and treatment options are largely dependent on clinicians’ personal preferences and experience (9). Therapeutic strategies currently reported in the literature include nonsteroidal anti-inflammatory drugs (NSAIDs), colchicine, glucocorticoids, conventional disease modifying anti-rheumatic drugs (DMARDs) and biological DMARDs (10). However, treatment effects are variable and there is a lack of randomized controlled trials (RCT) and large cohort studies to provide strong support for approaches in the management of PR (1). Methotrexate (MTX) and hydroxychloroquine (HCQ) are the mainstays of existing treatment, but some patients have poor results and certain side effects (11–13). This gap in knowledge necessitates a deeper exploration of more effective and safer therapeutic drugs.

Recent decades have witnessed the approval of a novel synthetic small molecule DMARDs, iguratimod (IGU), for the treatment of rheumatoid arthritis. Numerous clinical studies have reported the efficacy and safety of IGU monotherapy, in combination with other DMARDs and as an add-on therapy for RA (14). A recent Meta-analysis has shown its masterful ability in the treatment of inflammatory arthritis such as ankylosing spondylitis and degenerative arthritis in addition to RA (15). From this we hypothesized that iguratimod may serve as a potentially effective therapeutic option in the management of PR which is also an inflammatory arthritis. With few studies focusing on iguratimod for the treatment of PR, its efficacy for PR remains shrouded in mystery. Therefore, our study included patients with PR who had switched to IGU after insufficient response to MTX or HCQ to further analyze the efficacy and safety of IGU in the treatment of refractory PR.

## 2 Materials and methods

### 2.1 Patients and study design

**Figure 1:**
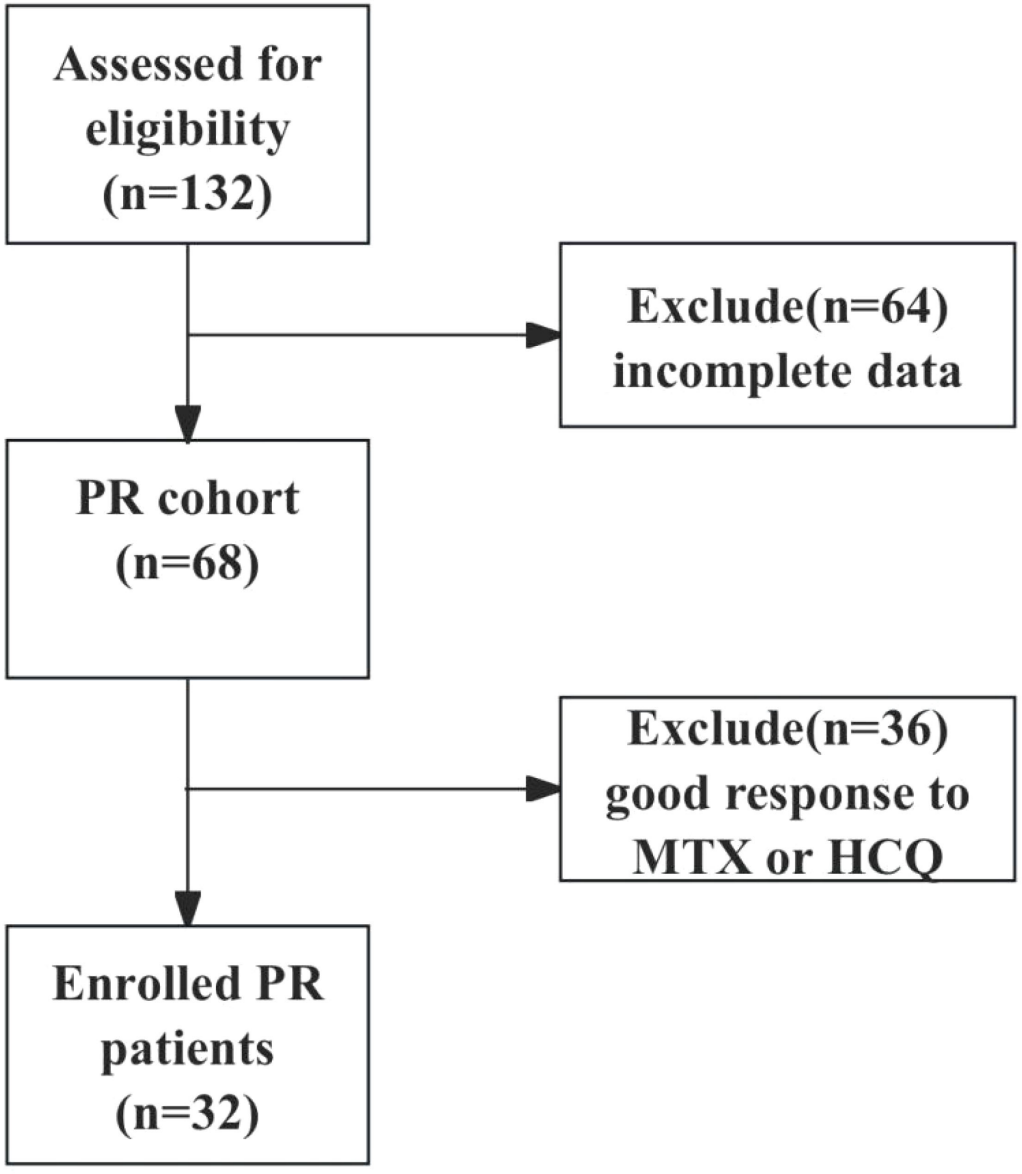
Flowchart of patient enrollment.

This retrospective study included patients with PR who attended the First People’s Hospital of Wenling between January 2019 and September 2023. Senior rheumatologists used the same inclusion criteria to diagnose and recruit patients with PR who met the criteria of Weisman (16) and all 3 additional criteria: 1) ≥18 years old; 2) seronegative for rheumatoid factor (RF) and anti-CCP antibodies; 3) medication history of IGU, MTX and (or) HCQ. Exclusion criteria were infection, cancer, connective tissue diseases, or other acute monoarthritides. The clinical data of the patients were further collected based on the electronic medical record and telephone follow-up visit, 64 patients with incomplete data were excluded, 36 patients with good response to MTX or HCQ were excluded, and 32 patients with poor response to MTX or HCQ (≥3 episodes in 3 months) were finally enrolled, and were switched to the treatment of IGU 25 mg twice daily or IGU 25 mg twice daily combined with MTX 10 mg weekly. The study was followed up until September 30th, 2024. The flowchart of patient enrollment is shown in **Figure 1**. The study was approved by the Ethics Committee of First People’s Hospital of Wenling (Ethics approval number: KY-2024-2031-01).

### 2.2 Data collection

We collected clinical data from patients based on electronic medical records and telephone follow-up: general demographic information, disease duration, affected joints, history of previous medications, number of attacks, frequency of attacks, duration of each attack, laboratory tests (erythrocyte sedimentation rate, C-reactive protein, blood counts, glutamic pyruvic transaminase values, glutamic oxaloacetic transaminase values, serum creatinine level, RF, and anti-CCP antibodies) before and after IGU treatment, as well as adverse events and prognosis. Recurrent episodes of swelling and pain in the joints are the most prominent and characteristic clinical manifestation of PR, and there is a lack of more objective criteria to assess disease activity. Drawing on previous research and clinical practice, we defined complete remission as no attacks for 3 months, partial remission as at least 50% reduction in the number of attacks for 3 months and no remission as less than 50% reduction in the number of attacks for 3 months, or progression to other rheumatic diseases such as RA (17). Flare was defined as ≥1 episodes per month despite 3 months of IGU treatment.

### 2.3 Statistical analysis

Data were processed and analyzed using GraphPad Prism10.1.1 software. Categorical variables were expressed as frequencies and percentages. Quantitative variables were described as mean ± standard deviation when normally distributed and median (interquartile range) when not normally distributed. The differences in the number of attacks and the duration of each attack before and after treatment with IGU were analyzed by paired t test and the difference in the frequency of attacks before and after treatment with IGU was analyzed by Wilcoxon matched-pairs signed rank test. Kaplan–Meier curve and Log-rank test were conducted to analyze the difference in time to achieve remission between IGU monotherapy group and IGU combined with MTX group. Statistical significance was set at P < 0.05.

## 3 Results

### 3.1 Baseline characteristics of patients with PR

The baseline demographic and clinical characteristics of the enrolled patients are shown in **Table 1**. The patients were predominantly male, with a mean age of 44.2 years and a median disease duration of 4.0 years. The affected joints included all the joints of the whole body, among which the metacarpophalangeal joints, wrist joints and elbow joints were involved in a higher proportion. Most patients did not have significantly elevated erythrocyte sedimentation rate and C-reactive protein level. In addition to MTX and HCQ, most patients had taken NSAIDs during previous episodes.

**Table 1:** Baseline characteristics of patients with palindromic rheumatism (N = 32).

Characteristics	
Age, mean ± SD, y	44.2 ± 13.5
Female, n (%)	9 (28.1)
Duration, median (IQR), y	4.0 (1.3, 8.8)
Involved structures	
Hand PIP joints, n (%)	9 (28.1)
MCP joints, n (%)	14 (43.8)
Wrists, n (%)	14 (43.8)
Elbows, n (%)	15 (46.9)
Shoulders, n (%)	11 (34.4)
Knees, n (%)	14 (43.8)
Ankles, n (%)	6 (18.8)
Foot joints, n (%)	7 (21.9)
ESR, mm/h, median (IQR)	11.5 (6.3, 21.5)
CRP, mg/L, median (IQR)	5.7 (3.7, 8.4)
Medications	
MTX, n (%)	26 (81.3)
HCQ, n (%)	18 (56.3)
NSAIDs, n (%)	20 (62.5)

PIP, proximal interphalangeal; MCP, metacarpophalangeal; ESR, erythrocyte sedimentation rate; CRP, C-reactive protein; MTX, methotrexate; HCQ, hydroxychloroquine; NSAIDs, non-steroidal anti-inflammatory drugs.

### 3.2 Effect of IGU on clinical parameters of patients with PR

**Figure 2:**
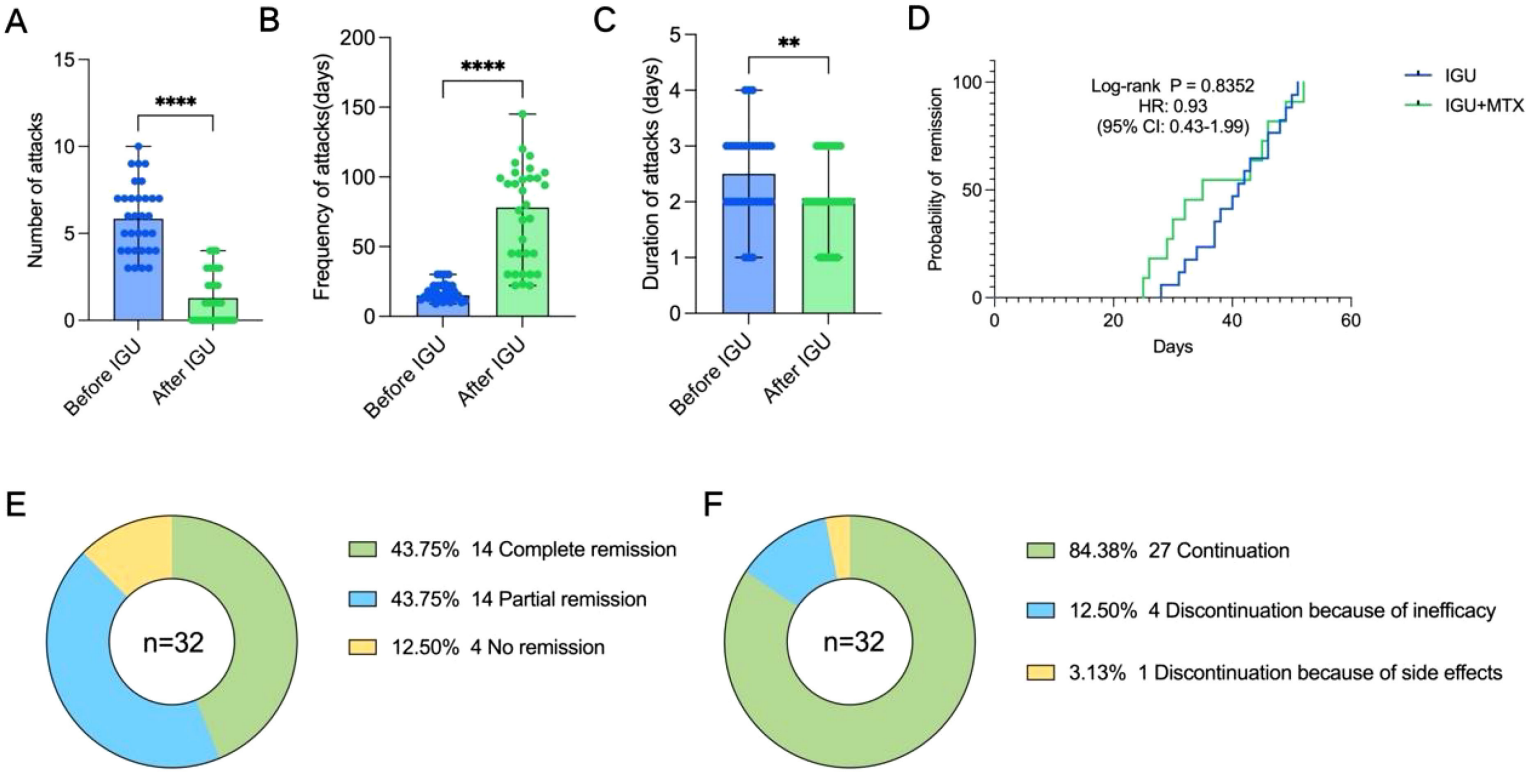
Effect of IGU on patients with PR. **(A)** The number of attacks over 3months before and after treatment with IGU. **(B)** The frequency of attacks before and after treatment with IGU. **(C)** The duration of each attack before and after treatment with IGU. **(D)** Kaplan–Meier curve displaying the difference in time to achieve remission between the IGU monotherapy group and the IGU combined with MTX group. **(E)** Remission status. **(F)** Continuity of IGU. (** P < 0.01, **** P < 0.0001).

The treatment regimens of the enrolled patients were divided into two groups, including the IGU monotherapy group and the IGU combined with MTX group (**Table 2**). The median duration of treatment with IGU was 11.3 months. The number of attacks for 3 months was significantly reduced after treatment with IGU compared with the previous period (1.3 ± 1.4 vs 5.8 ± 2.0, P<0.0001) (**Figure 2A**). The frequency of episodes was reduced and the duration of remission was prolonged after treatment with IGU (78.0(33.8,99.0) days vs 15.0(13.0,22.0) days, P <0.0001) (**Figure 2B**). The duration of each attack was shortened (2.1 ± 0.7 days vs 2.5 ± 0.8 days, P=0.0042) (**Figure 2C**).

**Table 2:** Effect of IGU on patients with palindromic rheumatism (N = 32).

Characteristics	
Medications	
IGU, n (%)	19 (59.4)
IGU+MTX, n (%)	13 (40.6)
Duration of treatment with IGU, median (IQR) month	11.3 (7.1, 17.8)
Time to remission, mean ± SD, day	39.5 ± 8.2
Flare, n (%)	8 (25)
Progression to RA, n	0

IGU, iguratimod; MTX, methotrexate; RA, rheumatoid arthritis.

We found no statistical difference in the time to reach remission (both complete and partial) between the IGU monotherapy group and the IGU combined with MTX group via survival analysis (P = 0.8352, HR: 0.93, 95% CI: 0.43-1.99) (**Figure 2D**), and the latter group appeared to reach remission faster in the early stage of treatment, but overall, there was no statistical difference. In this study, we found that 43.75% of patients reached complete remission and 43.75% reached partial remission after treatment with IGU (**Figure 2E**), and the time to reach remission was 39.5 ± 8.2 days (**Table 2**). Eight of the patients suffered flare after treatment, but no patient progressed to rheumatoid arthritis during the follow-up period (**Table 2**). 84.38% of the patients were able to have regular follow-ups and continued to use IGU, while 12.50% of the patients discontinued due to lack of efficacy, and 3.13% of the patients discontinued due to an adverse event (gastric upset) (**Figure 2F**). Regular hepatic and hematologic monitoring were conducted and the levels were all within normal limits. Overall, iguratimod has a favorable efficacy and safety profile in the treatment of PR.

## 4 Discussion

The study population included in this study was patients with PR who were seronegative for RF and anti-CCP antibodies. In Western countries, 39%-68% of patients with PR are positive for RF and anti-CCP antibodies, with a larger proportion developing rheumatoid arthritis (18). However, the frequency of RF or anti-CCP antibody positivity was much lower in the Asian PR cohort (<15%), with only 12.9% of patients developing RA during long-term follow-up (2, 19, 20). From the above studies, it can be found that RF and anti-CCP seronegative PR patients are less likely to progress to RA. A study enrolled 154 patients with early RA and found that 65 (42%) patients had a history of episodic joint pain and swelling, which occurred 6 months or more before the diagnosis of early RA in half of these patients (21). It is difficult to distinguish patients with PR who are positive for RF and anti-CCP antibodies from early RA, and we argue that this group of patients may be somehow more appropriately diagnosed as early RA. Despite relentless scrutiny, the relationship between PR and early RA is still inconclusive (18). Consequently, we focused on seronegative patients with PR and no patient progressed to RA in our study. This suggests that future studies may need to differentiate between RF and anti-CCP positive and negative patients with PR, and that there may be some differences in genetics and immunology between the two groups. Besides, it may be necessary to carry out a large-scale clinical study to redefine the diagnostic criteria for PR.

We found that patients with PR had significantly fewer attacks, longer remission time and shorter duration of each attack after treatment with IGU, which demonstrates the effectiveness of IGU in the treatment of PR. A previous study yielded similar results (22). Despite common genetic and immune risk factors (1), the clinical features of PR differ from those of RA, and its pathogenesis may differ as well. It has been found that patients with PR are genetically susceptible, carrying mutations in the HLA-DRB1*0803 allele (23), and a high frequency of mutations in the MEFV, NLRP12 and TNFSFA genes have been identified (20, 24, 25), suggesting that both autoimmune and autoinflammatory profiles may be involved in the pathogenesis of PR. Iguratimod is a novel DMARDs with both anti-inflammatory and immunomodulatory effects (15), which can participate in the immune response by regulating CD4+ T cells and activated B lymphocytes (14), and exert anti-inflammatory effects by inhibiting the production of various cytokines, such as IL-1β, TNF-α, IL-6, IL-8, and monocyte chemotactic protein (MCP)-1 (15). We hypothesize that the dual mechanism of IGU is quite compatible with the pathogenesis of PR, and thus IGU is effective in patients with PR. However, given the lack of a control group such as HCQ+MTX in our study, we have to acknowledge that superiority of IGU over HCQ+MTX cannot be concluded from this design and future RCTs are needed to test this hypothesis.

In this study, we found no statistically significant difference in the time to achieve disease remission (both complete and partial remission) between the IGU monotherapy group and the IGU combined with MTX group. While there is limited clinical research on IGU for PR, studies of RA may provide valuable insights. A meta-analysis showed that both the IGU monotherapy group and the IGU+MTX group effectively reduced disease activity in RA (26). A 54-week study of RA patients found both groups demonstrated significant DAS28-CRP reductions compared to baseline, with comparable efficacy observed between the two groups (27). This study demonstrates the feasibility of controlling the disease through the use of minimal drug combinations, thereby reducing the potential adverse effects associated with polypharmacy. Given that methotrexate has side effects such as liver and kidney toxicity (28), IGU may serve as a better option in treatment of PR, which needs to be confirmed by larger samples in clinical studies.

There are some limitations in this study. First, small sample size due to the rarity of PR and the lack of well-accepted diagnostic criteria. Second, this is a retrospective study and no validated tool was used to assess disease activity and efficacy. Future development of more objective criteria for assessing disease activity based on more evidence-based medicine is needed. Third, this study was mainly focused on refractory PR, in the future, stratified management of patients with different disease states, and a more comprehensive study of the efficacy of IGU are of great necessity. Fourth, only paired comparisons were applied for evaluation the efficacy of IGU, absence of adjustment for prior DMARDs exposure or baseline attack frequency introduces bias. Multivariate analysis is necessary in future studies. There are very few studies on IGU treatment of PR and our study can provide a profound point for clinical practice and future research.

In conclusion, iguratimod has shown efficacy in improving symptoms in patients with PR who have not responded sufficiently to traditional DMARDs, with a favorable safety profile. We look forward to real-world studies or RCTs with larger samples to confirm the efficacy and safety of iguratimod in the treatment of PR and the development of formal diagnostic and response criteria for PR in future guidelines. Furthermore, inflammatory biomarkers (e.g., IL-6, MCP-1) exploration, long-term PR to RA progression tracking, and better outcome standardization are far-reaching directions for future research.

## Data Availability

The raw data supporting the conclusions of this article will be made available by the authors, without undue reservation.

## References

[1] Mankia K Emery P. Palindromic rheumatism as part of the rheumatoid arthritis continuum. Nat Rev Rheumatol. (2019) 15:687–95. doi: 10.1038/s41584-019-0308-5 31595059

[2] Russell AS Devani A Maksymowych WP. The role of anti-cyclic citrullinated peptide antibodies in predicting progression of palindromic rheumatism to rheumatoid arthritis. J Rheumatol. (2006) 33:1240–2. 16724377

[3] Maksymowych WP Suarez-Almazor ME Buenviaje H Cooper BL Degeus C Thompson M. HLA and cytokine gene polymorphisms in relation to occurrence of palindromic rheumatism and its progression to rheumatoid arthritis. J Rheumatol. (2002) 29:2319–26. 12415587

[4] Emad Y Anbar A Abo-Elyoun I El-Shaarawy N Al-Hanafi H Darwish H. In palindromic rheumatism, hand joint involvement and positive anti-CCP antibodies predict RA development after 1 year of follow-up. Clin Rheumatol. (2014) 33:791–7. doi: 10.1007/s10067-014-2569-3 24623460

[5] Koskinen E Hannonen P Sokka T. Palindromic rheumatism: longterm outcomes of 60 patients diagnosed in 1967-84. J Rheumatol. (2009) 36:1873–5. doi: 10.3899/jrheum.090025 19648311

[6] Mankia K Emery P. What can palindromic rheumatism tell us? Best Pract Res Clin Rheumatol. (2017) 31:90–8. doi: 10.1016/j.berh.2017.09.014 29221602

[7] Mankia K D’Agostino MA Wakefield RJ Nam JL Mahmood W Grainger AJ. Identification of a distinct imaging phenotype may improve the management of palindromic rheumatism. Ann Rheum Dis. (2019) 78:43–50. doi: 10.1136/annrheumdis-2018-214175 30297331

[8] Zheng C Wang F Sun Y Zhou Z You Y He D. Identification of distinct genetic profiles of palindromic rheumatism using whole-exome sequencing. Arthritis Rheumatol. (2023) 75:1947–57. doi: 10.1002/art.42614 37219934

[9] Corradini D Di Matteo A Emery P Mankia K. How should we treat palindromic rheumatism? A systematic literature review. Semin Arthritis Rheumatol. (2021) 51:266–77. doi: 10.1016/j.semarthrit.2020.11.008 33401055

[10] Kavandi H Hashemi SZ Khalesi E Khabbazi A. Treatment of palindromic rheumatism: A systematic review. Int J Clin Pract. (2021) 75:e14868. doi: 10.1111/ijcp.14868 34525234

[11] Ghassembaglou A Esalatmanesh K Gadakchi L Nourmohammadi F Khabbazi A. Long-term outcome of patients with palindromic rheumatism treated with methotrexate. Int J Rheum Dis. (2022) 25:489–95. doi: 10.1111/1756-185X.14302 35133068

[12] Padhan P Thakur B. Effect of low dose methotrexate as an add-on therapy in patients with palindromic rheumatism unresponsive to hydroxychloroquine: An observational study. Eur J Rheumatol. (2021) 8:130–2. doi: 10.5152/eurjrheum.2021.20062 PMC9770407 34101572

[13] Hannonen P Möttönen T Oka M. Palindromic rheumatism. A clinical survey of sixty patients. Scand J Rheumatol. (1987) 16:413–20. doi: 10.3109/03009748709165412 3423751

[14] Xie S Li S Tian J Li F. Iguratimod as a new drug for rheumatoid arthritis: current landscape. Front Pharmacol. (2020) 11:73. doi: 10.3389/fphar.2020.00073 32174824 PMC7054862

[15] Long Z Zeng L Yang K Chen J Luo Y Dai CC. A systematic review and meta-analysis of the efficacy and safety of iguratimod in the treatment of inflammatory arthritis and degenerative arthritis. Front Pharmacol. (2024) 15:1440584. doi: 10.3389/fphar.2024.1440584 39449973 PMC11499590

[16] Guerne PA Weisman MH. Palindromic rheumatism: part of or apart from the spectrum of rheumatoid arthritis. Am J Med. (1992) 93:451–60. doi: 10.1016/0002-9343(92)90177-d 1341421

[17] Sadri M Esalatmanesh K Khabbazi A. Efficacy of leflunomide in the treatment of palindromic rheumatism. Int J Rheum Dis. (2022) 25:893–6. doi: 10.1111/1756-185X.14364 35666009

[18] Sanmarti R Frade-Sosa B Morla R Castellanos-Moreira R Cabrera-Villalba S Ramirez J. Palindromic rheumatism: just a pre-rheumatoid stage or something else? Front Med (Lausanne). (2021) 8:657983. doi: 10.3389/fmed.2021.657983 33842513 PMC8026891

[19] Chen HH Chao WC Liao TL Lin CH Chen DY. Risk of autoimmune rheumatic diseases in patients with palindromic rheumatism: A nationwide, population-based, cohort study. PloS One. (2018) 13:e0201340. doi: 10.1371/journal.pone.0201340 30048527 PMC6062130

[20] Lu LY Keng HM Chu JJ Lin XT Hsu CM Sung PK. TNF receptor I polymorphism is associated with persistent palindromic rheumatism. Scand J Rheumatol. (2007) 36:278–84. doi: 10.1080/03009740701286805 17763205

[21] Ellingwood L Schieir O Valois MF Bartlett SJ Bessette L Boire G. Palindromic rheumatism frequently precedes early rheumatoid arthritis: results from an incident cohort. ACR Open Rheumatol. (2019) 1:614–9. doi: 10.1002/acr2.11086 PMC6917323 31872182

[22] Yuan F He J Luo J Zhang X Lin J Chen Y. Iguratimod efficacy in palindromic rheumatism treatment. Immun Inflammation Dis. (2023) 11:e932. doi: 10.1002/iid3.932 PMC10303674 37382250

[23] Kim SK Lee HS Lee KW Bae SC Jun JB. Palindromic rheumatism: different genetic background implies a distinct disease entity. Ann Rheum Dis. (2006) 65:1539–40. doi: 10.1136/ard.2006.052928 PMC1798353 17038462

[24] Cuervo A Sanmartí R Ramírez J Castellanos-Moreira R Inciarte-Mundo J Aróstegui JI. Palindromic rheumatism: Evidence of four subtypes of palindromic-like arthritis based in either MEFV or rheumatoid factor/ACPA status. Joint Bone Spine. (2021) 88:105235. doi: 10.1016/j.jbspin.2021.105235 34098104

[25] Wang J Zhang Q Xu L Lv C Liu R Zhang M. A case of episodic and refractory arthritis due to a novel variant of NLRP12. Ann Rheum Dis. (2022) 81:e33. doi: 10.1136/annrheumdis-2020-217023 32066557

[26] Ouyang D Ma YZ Zou J Wang YL Chen Z Yang YY. Effectiveness and safety of iguratimod monotherapy or combined with methotrexate in treating rheumatoid arthritis: A systematic review and meta-analysis. Front Pharmacol. (2022) 13:911810. doi: 10.3389/fphar.2022.911810 35991879 PMC9389904

[27] Inoue A Nozaki Y Hirooka Y Kinoshita K Chiba Y Funauchi M. The effectiveness and retention rate of iguratimod in Japanese rheumatoid arthritis patients with/without methotrexate in daily medical care. Life (Basel). (2020) 10:261. doi: 10.3390/life10110261 33138014 PMC7692096

[28] Wang W Zhou H Liu L. Side effects of methotrexate therapy for rheumatoid arthritis: A systematic review. Eur J Med Chem. (2018) 158:502–16. doi: 10.1016/j.ejmech.2018.09.027 30243154

